# Supplementary material for: RNA Interference Reveals the Impacts of CYP6CY7 on Imidacloprid Resistance in Aphis glycines
Source: Insects. 2024 Mar 13;15(3):188. doi: 10.3390/insects15030188 (PMC10970732; doi:10.3390/insects15030188)
Supplement: Supplementary file 1 [file insects-15-00188-s001.zip › insects-2831972-supplementary.pdf]

Table S1 Primers for cloning, qRT-PCR and RNA interference

Primer name	Primer sequences (5'-3')	usage
CYP6CY7-F	CAGTCACGCACGGATGTTCT	RT-PCR for cloning
CYP6CY7-R	GCCGTTTTTCGGACTGACG	
CYP6CY8-F	AGCTTTGCTAATATTTGGTTTGACT	
CYP6CY8-R	TCAGTTACATGACACTTGTTATTGTTT	
CYP6CY9-F	TGTTCGAGATAGCGACCAGC	
CYP6CY9-R	GCAATAGGGCATATATTAACGGGG	
CYP6CY12-F	ATACGTTGCAGTTTTCACGC	
CYP6CY12-R	GCCATTTTTGGCCACCATCAT	
CYP6CY14-F	TAAGCGTTCCCTGCGACAAT	
CYP6CY14-R	AAAGTGAGTACAGCGCCACA	
CYP6CY16-F	TGACGTACGATAAATGGCGGA	
CYP6CY16-R	TGTTCAAGACAGCTCGTCAA	
CYP6CY18-F	TGTATTTTTCATACAATGTTTGCGTCG	
CYP6CY18-R	TAGCCAAATACCATTTTCTGGCA	
CYP6CY20-F	TGCAAGCAATCTTGGACGATG	
CYP6CY20-R	AATGTTAGCCAAATTCCATGTTTTG	
CYP6CY48-F	ATTCATAAGAGAGATACACGTGGTG	
CYP6CY48-R	TTACAGGAATTACGGTTTTTTCACT	
CYP6CZ1-F	TATTGGTCACCGCGTTCCT	
CYP6CZ1-R	AATGGGTGAAATACCAATTCTCAG	
CYP6DB1-F	TCTGCCGCACCGCTATTATT	
CYP6DB1-R	TGGACCAGTTCCGAAGGGTA	
CYP6DD1-F	TTTCCAGCGGCCGTCATAAT	
CYP6DD1-R	GTGCGGTCTCAATGTGTTCG	
TBP-q-F	ATAGTATGATGCATTCACAAACGCC	qRT-PCR
TBP-q-R	TTGTAGCGCTGGTATAGTTGATGAT	
RPS9-q-F	TAAGCGTGAAGTATGGCGTGTGA	
RPS9-q-R	ATACACCAATGCGAACCAATCTACG	
CYP6CY7-q-F	GGTGTAGATACTCATCCGTCTGTCA	
CYP6CY7-q-R	GCTTCTGTCGCATCGTTCTCC	
CYP6CY8-q-F	GGCATAGAGCACCCAATAGACTTGT	
CYP6CY8-q-R	CGCATCTGAAACATACCTCCGTACT	
CYP6CY9-q-F	AATAGCATACGCAGGAATCAGAAC	
CYP6CY9-q-R	AAGCAACGAAGGATTACCAAGTG	
CYP6CY12-q-F	TGTCACTTATGTCGCCAAGATTG	
CYP6CY12-q-R	CTATGCCGTTCGCCTCTCTG	
CYP6CY14-q-F	GTATTATCCAAATCCCGACACTTTC	
CYP6CY14-q-R	ACGAGGTCCATCTCCAAACG	
CYP6CY16-q-F	AAGGCACTTACTTGGCATTTGG	
CYP6CY16-q-R	TGTTGACGGGAATTTCGGTTTG	
CYP6CY18-q-F	TTGAGCCCAGCGTTCACTTCAG	
CYP6CY18-q-R	TTTGCCCAACATATCTCGCACTTCT	
CYP6CY20-q-F	GCTCTGTTGCTGTGGATCGCT	
CYP6CY20-q-R	GATACGCACACGACTCGGCA	
CYP6CY48-q-F	AGTAAGCCGTTTCCGTTATTCG	
CYP6CY48-q-R	TGACCAGAGAACTCGTTGTAGAT	
CYP6CZ1-q-F	CCAGTACACGCCATTCACTACGA	
CYP6CZ1-q-R	GGACCATCTCCGAACGGCATAT	
CYP6DB1-q-F	GTAATGCTAAGGACCGTGTTCT	
CYP6DB1-q-R	ATGATGCTCGGCGACCAA	
CYP6DD1-q-F	CGCACCGTATAGACCATCCG	
CYP6DD1-q-R	GGTTCCAATCACAGCACTCG	
siRNA of CYP6CY7	GCACGGCAAUAUUUCAUAUTT	RNA interference
	AUAUGAAAUAUUGCCGUGCTT	
Negative control	UUCUCCGAACGUGUCACGUTT	
	ACGUGACACGUUCGGAGAATT	
